# Association between the PSCA rs2976392 polymorphism and susceptibility to gastric cancer: a meta-analysis

**DOI:** 10.3389/fgene.2025.1618597

**Published:** 2025-07-16

**Authors:** Xiao-Hua Lin, Da-Jun Chen, Li-Juan Wang, Xiu-Ping Wan

**Affiliations:** Department of Gastroenterology, The Quzhou Affiliated Hospital of Wenzhou Medical University, Quzhou People’s Hospital, Quzhou, China

**Keywords:** PSCA, rs2976392, polymorphism, gastric cancer, meta-analysis

## Abstract

**Background:**

Genetic polymorphisms, such as PSCA rs2976392, have been implicated in gastric carcinogenesis, but it is unclear whether there is a direct association. Thus, we conducted a comprehensive meta-analysis to evaluate the association between the PSCA rs2976392 polymorphism and susceptibility to gastric cancer (GC).

**Methods:**

A systematic search of the PubMed, Web of Science, Embase, and Cochrane Library databases was performed up to 13 March 2025. Pooled odds ratios (ORs) with 95% confidence intervals (CIs) were calculated for five genetic models. Subgroup analyses were conducted according to ethnicity and Hardy–Weinberg equilibrium (HWE) status. Heterogeneity and publication bias were assessed, and sensitivity analyses were performed.

**Results:**

A total of 13 studies involving 9,255 patients with gastric cancer and 8,903 controls were included. Overall, a significant association between the PSCA rs2976392 polymorphism and increased GC risk was observed under the allele model (OR = 1.29, 95% CI: 1.19–1.40), dominant model (OR = 1.53, 95% CI: 1.3–1.77), homozygous model (OR = 1.52, 95% CI: 1.18–1.96), and heterozygous model (OR = 1.52, 95% CI: 1.33–1.73), but not under the recessive model. Subgroup analyses revealed a strong association in Asian populations with all genetic models, whereas Caucasians showed a significant association only with the homozygous model. No significant publication bias was detected, and sensitivity analyses confirmed the robustness of the results.

**Conclusion:**

This meta-analysis provides strong evidence that the PSCA rs2976392 AA genotype significantly increases susceptibility to gastric cancer, particularly among Asians.

## Introduction

Gastric cancer (GC) is one of the most common malignancies worldwide and represents one of the four leading causes of cancer-related deaths (Siegel et al., 2025). Although the incidence and mortality rates of GC have markedly decreased in some countries, GC remains a major public health challenge because of its late diagnosis and poor prognosis (Thrift et al., 2023). The development of gastric cancer is a multifactorial and multistep process influenced by both environmental and genetic factors. Well-established environmental risk factors include *Helicobacter pylori* infection, dietary habits, smoking, and alcohol consumption (Maubach et al., 2025; Wizenty and Sigal, 2025). However, there is growing evidence that genetic susceptibility factors contribute to the occurrence and development of GC.

Genome-wide association studies (GWASs) have revealed numerous single nucleotide polymorphisms (SNPs) associated with gastric cancer susceptibility (Wang et al., 2017). Among them, polymorphisms in the prostate stem cell antigen (PSCA) gene have attracted increasing attention (Hu et al., 2016). The PSCA gene, located on chromosome 8q24.2, encodes a glycosylphosphatidylinositol-anchored cell surface protein that is involved in cell adhesion, proliferation, and differentiation (Lao-Sirieix et al., 2010). PSCA expression has been found to be downregulated in gastric cancer tissues compared with adjacent normal tissues, suggesting a potential tumor suppressor role in gastric cancer (Xu et al., 2020).

One particular SNP, rs2976392, which is located in the 5′-UTR of the PSCA gene, has been identified as a possible functional variant affecting PSCA transcriptional activity (Lu et al., 2010). This polymorphism may functionally influence PSCA expression levels, thereby contributing to individual differences in cancer susceptibility (Study Group of Millennium Genome Project for Cancer et al., 2008; Merrell et al., 2011). Several studies indicate that the A allele of rs2976392 is associated with an increased risk of gastric cancer (Matsuo et al., 2009; Ou et al., 2010; Kupcinskas et al., 2014; Cai et al., 2017; Dantas et al., 2020), possibly through altered gene expression and increased susceptibility of gastric mucosal cells to malignant transformation, but the results are conflicting and inconclusive.

Many studies indicate an association between PSCA rs2976392 polymorphism and GC risk (Matsuo et al., 2009; Lu et al., 2010; Ou et al., 2010; Kupcinskas et al., 2014; Qiu et al., 2016; Cai et al., 2017; Dantas et al., 2020). However, this association remains a subject of controversy. Therefore, the aim of the present study is to synthesize data from case-control studies to comprehensively evaluate the association between PSCA rs2976392 polymorphism and GC susceptibility.

## Materials and methods

### Search strategy

The current study was conducted according to the Preferred Reporting Items for Systematic Reviews and Meta-analysis (PRISMA) guidelines (Page et al., 2021). A comprehensive literature search on PubMed, Web of Science, Cochrane Library, and Embase databases to acquire relevant studies about the association of PSCA rs2976392 polymorphism and susceptibility to gastric cancer up to 13 March 2025. The combination of the following keywords and terms was used: “PSCA”, “rs2976392”, “polymorphism”, “SNP”, and “gastric cancer”. Reference lists of retrieved articles were manually searched to identify suitable studies.

### Inclusion and exclusion criteria

Available studies were included based on the following criteria: (1) case-control or cohort studies; (2) examined the association of the PSCA rs2976392 polymorphism with gastric cancer risk; (3) provided sufficient data to calculate odds ratio (OR) with a 95% confidence interval. Exclusion criteria included: (1) duplicated or overlapping data; (2) reviews, meta-analyses, case reports, and conference summaries; (3) insufficient data to analyze.

### Data extraction and quality assessment

Two investigators independently searched, assessed, and extracted data based on predefined inclusion and exclusion criteria. Disagreements were resolved by discussion or consultation with a third reviewer. The following information was extracted: first author, publication year, country, ethnicity, sample size, genotype frequencies, and Hardy-Weinberg equilibrium (HWE) status in controls. The quality of studies was assessed using the Newcastle-Ottawa Scale (NOS), and studies scoring ≥6 were considered high-quality.

### Statistical analysis

The strength of the association of PSCA rs2976392 polymorphism with gastric cancer risk was examined by odd ratios (ORs) with 95% confidence intervals (CIs) under five genetic models: allele model (A vs. G), dominant model (GA + AA vs. GG), recessive model (AA vs. GA + GG), homozygous model (AA vs. GG), and heterozygous model (GA vs. GG). Hardy-Weinberg equilibrium (HWE) assessed by chi-square test. Between-studies heterogeneity was assessed with Cochran’s Q and the I^2^ statistics. Fixed-effects models (Mantel–Haenszel) were used when I^2^ < 50%, otherwise random-effects models (DerSimonian–Laird) were applied. Subgroup analyses were conducted by stratification of ethnicity and HWE test to identify potential sources of heterogeneity. Publication bias was evaluated using Begg’s funnel plots and Egger’s test. All the statistical analyses were performed using STATA 12.0 (StataCorp., T.X., United States).

## Results

### Study selection and study characteristics

**FIGURE 1:**
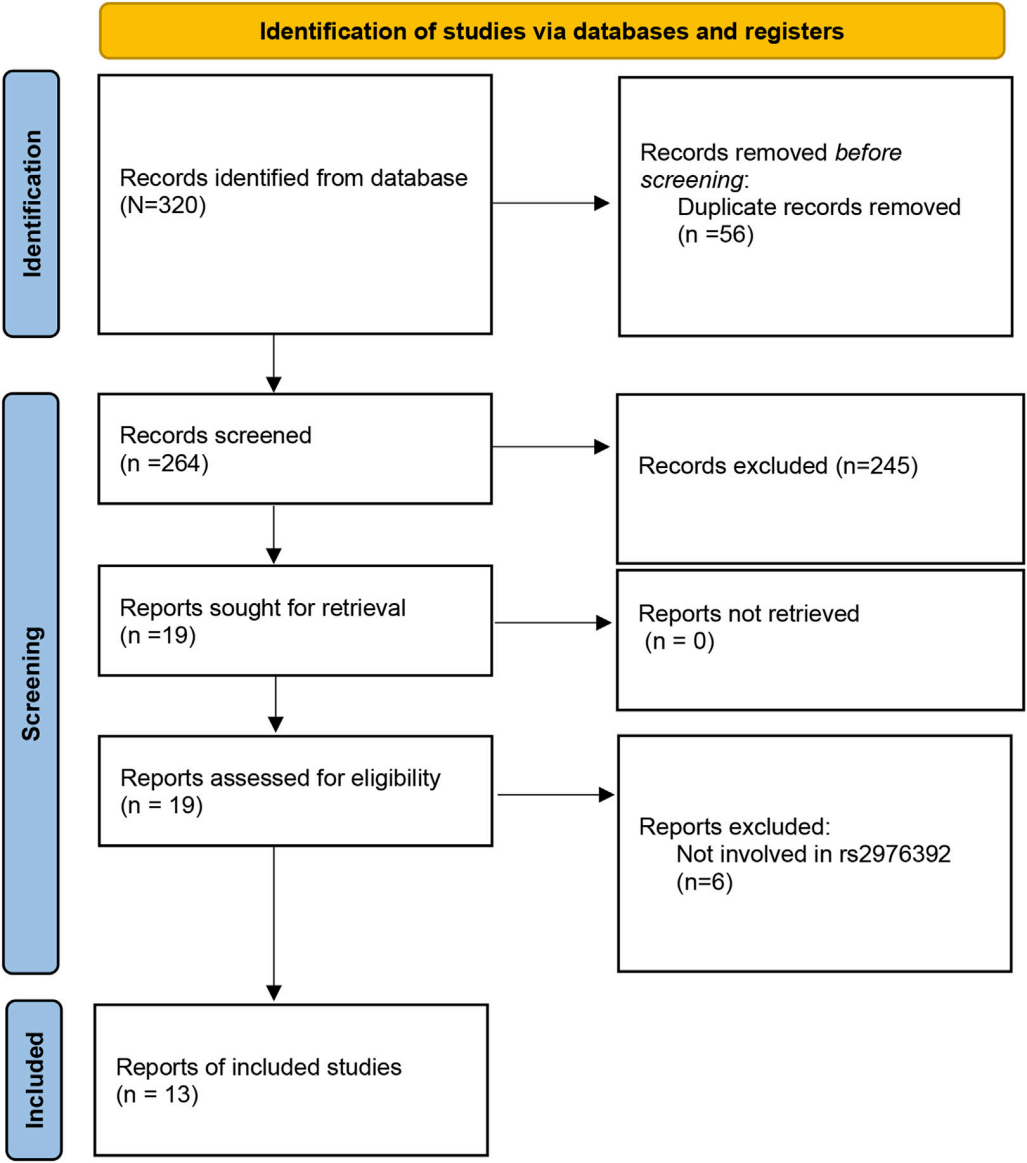
Flow diagram of the literature search.

The initial search of the PubMed, Web of Science, Cochrane Library, and Embase databases identified 320 potential studies. Among them, 56 duplicate studies were then removed, leaving 141 studies for further selection. Among these articles, 245 studies were excluded due to screening of the abstract and title. After full-text screening, 6 papers were excluded due to irrelevant reports. Finally, a total of 13 studies (Study Group of Millennium Genome Project for Cancer et al., 2008; Matsuo et al., 2009; Wu et al., 2009; Lu et al., 2010; Ou et al., 2010; Kupcinskas et al., 2014; Sun et al., 2015; Zhang et al., 2015; Qiu et al., 2016; Turdikulova et al., 2016; Cai et al., 2017; Yan et al., 2019; Dantas et al., 2020) involving 9,255 cases and 8,903 controls were included in the meta-analysis (Figure 1). The publication year of the studies ranged from 2008 to 2020. The countries of these studies included Japan, China, Brazil, Uzbekistan, Latvia, Japan, and Korea. The participants included Caucasians and Asians population. The genotype distribution in the control group for 3 studies was not consistent with HWE. The Newcastle-Ottawa Scale (NOS) scores are shown in Table 2, with the scores of the included studies ranged from six to eight (Table 1). The Kappa’s score for data extraction was 0.831, indicating good interobserver agreement between the two investigators.

**TABLE 1:** The main characteristics of the included studies.

Author	Year	Country	Ethnicity	Case			Control			Sample size		HWE (control)	NOS score
				GG	GA	AA	GG	GA	AA	Case	Control		
Yan	2019	China	Asian	267	236	46	349	192	51	549	592	0.001	7
Cai	2017	China	Asian	213	223	45	268	173	47	481	488	0.016	7
Zhang	2015	China	Asian	190	208	38	231	184	36	436	451	0.939	6
Wu	2009	China	Asian	453	480	85	492	429	81	1,018	1,002	0.350	6
Qiu	2016	China	Asian	535	488	101	682	388	122	1,124	1,192	0.001	6
Sun	2015	China	Asian	319	308	65	403	299	72	692	774	0.130	6
Lu	2010	China	Asian	500	464	79	602	402	78	1,043	1,082	0.336	6
Ou	2010	China	Asian	99	85	12	130	102	14	196	246	0.298	7
Dantas	2020	Brazil	Caucasian	11	66	25	19	50	33	102	102	0.994	6
Turdikulova	2016	Uzbekistan	Caucasian	87	107	74	87	114	47	268	248	0.377	7
Kupcinskas	2014	Latvia	Caucasian	34	113	102	62	116	54	249	232	0.985	7
Matsuo	2009	Japan	Asian	331	328	48	274	337	96	707	707	0.635	8
Sakamoto a	2008	Korea	Asian	134	453	278	122	175	93	865	390	0.053	8
Sakamoto b	2008	Japan	Asian	97	691	737	211	650	536	1,525	1,397	0.545	8

Hp, Haptoglobin; HWE, Hardy–Weinberg equilibrium.

### Quantitative synthesis

**FIGURE 2:**
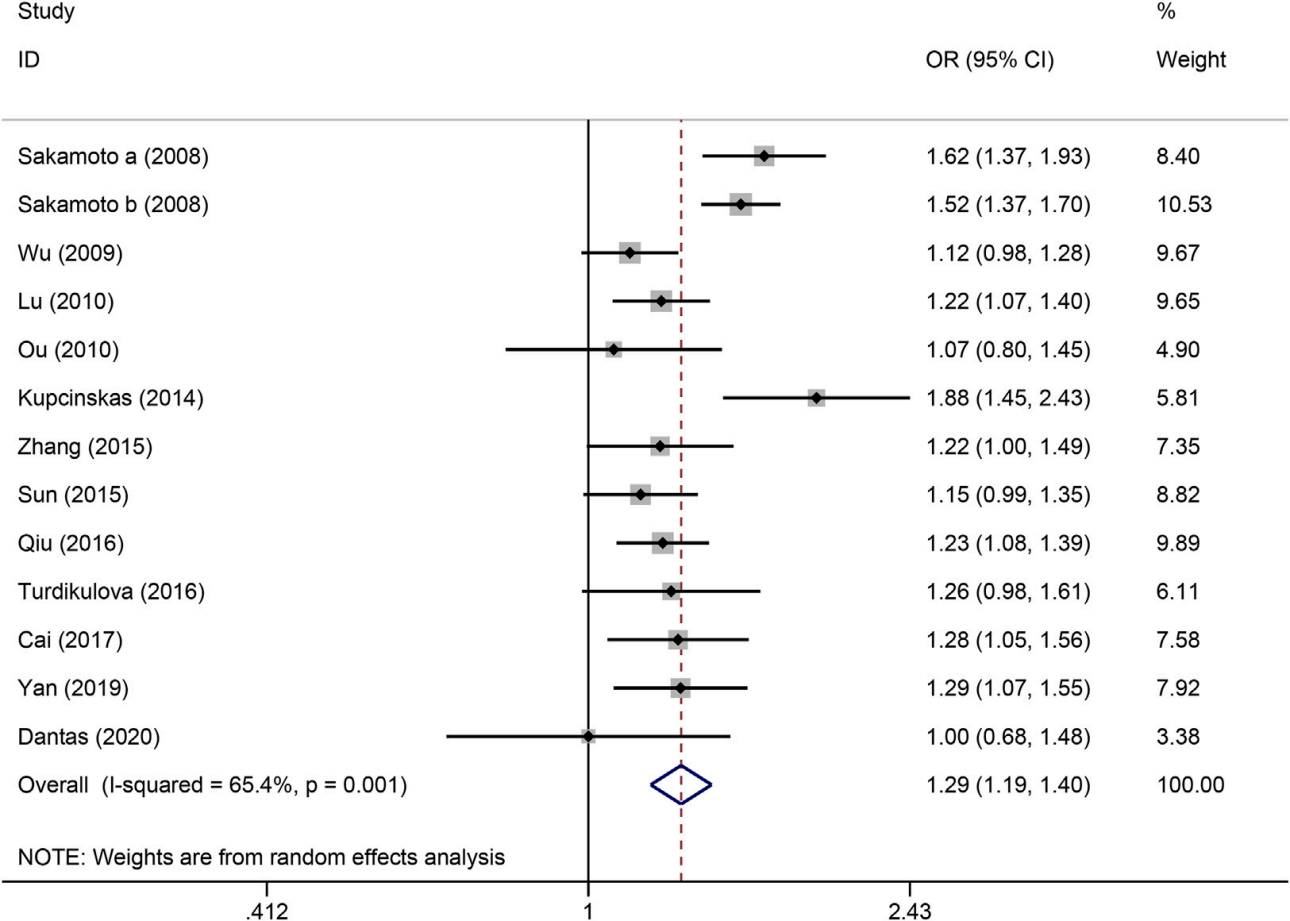
Forest plot of the correlation between PSCA rs2976392 polymorphism and gastric cancer in allele model (A vs. G).

**FIGURE 3:**
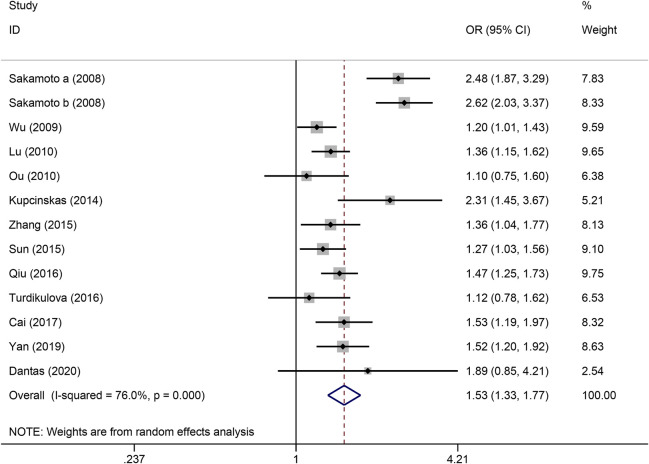
Forest plot of the correlation between PSCA rs2976392 polymorphism and gastric cancer in dominant model (GA + AA vs. GG).

**FIGURE 4:**
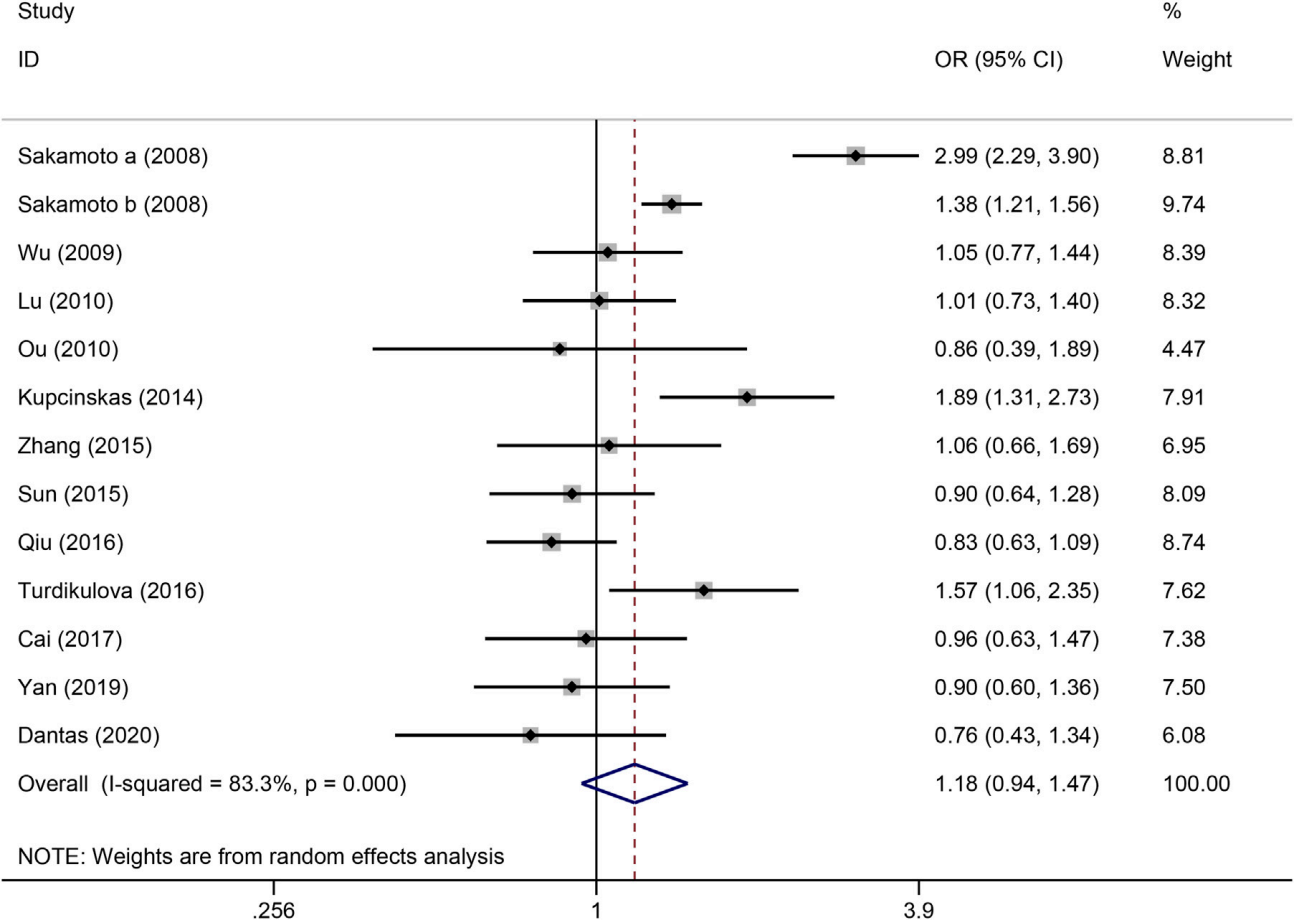
Forest plot of the correlation between PSCA rs2976392 polymorphism and gastric cancer in recessive model (AA vs. GA + GG).

**FIGURE 5:**
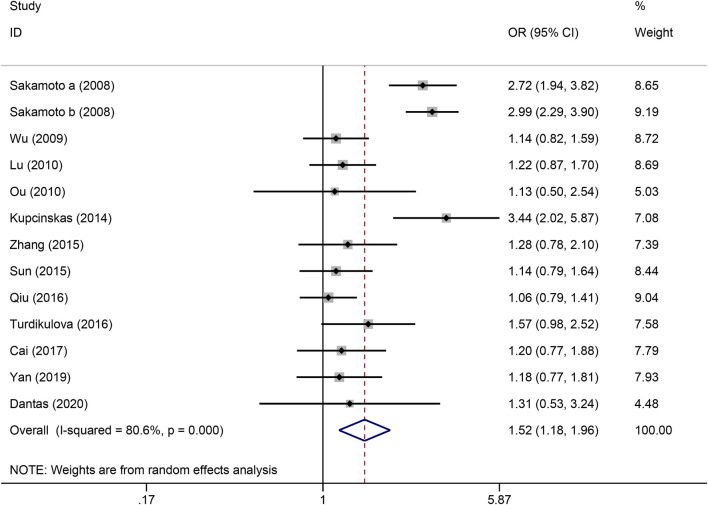
Forest plot of the correlation between PSCA rs2976392 polymorphism and gastric cancer in homozygous model (AA vs. GG).

**FIGURE 6:**
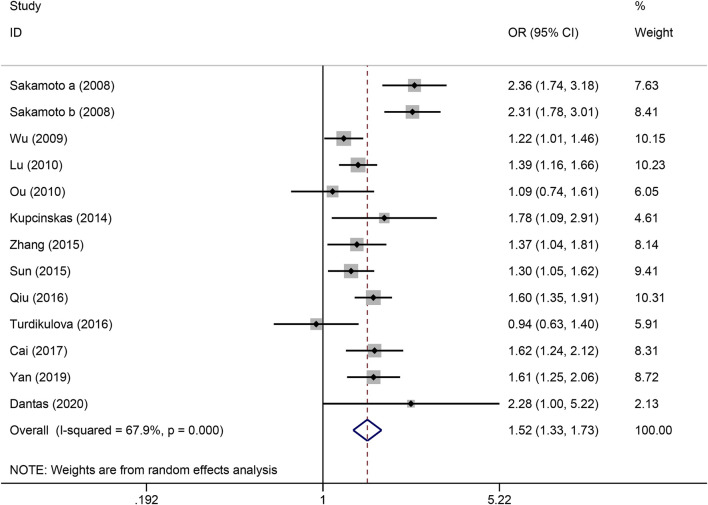
Forest plot of the correlation between PSCA rs2976392 polymorphism and gastric cancer in heterozygous model (GA vs. GG).

The strength of the association between the PSCA rs2976392 polymorphism and gastric cancer risk is presented in Table 2. A significant association was observed under the allele model (A vs. G), with a pooled OR of 1.29 (95% CI: 1.19–1.40; P = 0.001), indicating that the A allele is associated with increased GC risk (Figure 2). In the dominant model, individuals carrying A allele showed a significantly higher risk of gastric cancer compared to GG homozygotes, with a pooled OR of 1.53 (95% CI: 1.33–1.77, P = 0.001) (Figure 3). A stronger association was detected in the homozygous comparison (AA vs. GG), where the pooled OR was 1.52 (95% CI: 1.18–1.96, P = 0.001) (Figure 4). In the heterozygous model, individuals with the GA genotype also exhibited an increased risk compared to GG homozygotes (OR = 1.52, 95% CI = 1.33–1.73, P = 0.001) (Figure 5), but no association was observed in the recessive model (OR = 1.18, 95% CI = 0.94–1.47, P = 0.157) (Figure 6). Subgroup analyses were carried out according to ethnicity, we found a significant association between PSCA rs2976392 polymorphism and GC risk among the Asian population under the four genetic model (GA + AA vs. GG: OR = 1.52, 95%CI: 1.30–1.78, P = 0.001; AA vs. GG: OR = 1.42, 95%CI: 1.07–1.89, P = 0.016; GA vs. GG: OR = 1.54, 95%CI: 1.34–1.76, P = 0.001; A vs. G: OR = 1.28, 95%CI: 1.17–1.39, P = 0.001), and only associated with the Caucasian population under the homozygous genetic model (AA vs. GG: OR = 2.01, 95%CI: 1.11–3.65, P = 0.022). Moreover, after stratified by HWE test, a significant correlation was observed in the subgroup of HWE >0.05 under allele model, dominant model, homozygous model, and heterozygous model, but not in the recessive model (Table 2).

**TABLE 2:** Summary of meta-analysis of association of PSCA rs2976392 polymorphism and GC risk.

Model	Group	Studies	Overall effect			Heterogeneity	
			OR (95% CI)	*Z*-score	*p*-value	*I* ^2^ (%)	*p*-value
AA vs. GG + GA	Overall	13	1.18 (0.94, 1.47)	1.42	0.157	83.3	0.001
	Caucasian	3	1.37 (0.85, 2.21)	0.42	0.676	71.6	0.030
	Asian	10	1.13 (0.87, 1.46)	2.09	0.036	85.8	0.001
	HWE >0.05	10	1.28 (0.99, 1.66)	1.90	0.058	83.5	0.001
	HWE <0.05	3	0.87 (0.71, 1.07)	1.32	0.186	0	0.840
GA + AA vs. GG	Overall	13	1.53 (1.33, 1.77)	5.78	0.001	76	0.001
	Caucasian	3	1.64 (0.98, 2.75)	1.90	0.058	66.7	0.050
	Asian	11	1.52 (1.30, 1.78)	5.28	0.001	79.5	0.001
	HWE >0.05	11	1.55 (1.27, 1.91)	4.22	0.001	81.9	0.001
	HWE <0.05	3	1.50 (1.33, 1.69)	6.66	0.001	0	0.958
AA vs. GG	Overall	13	1.52 (1.18, 1.96)	3.19	0.001	80.6	0.001
	Caucasian	3	2.01 (1.11, 3.65)	2.29	0.022	65.5	0.055
	Asian	11	1.42 (1.07, 1.89)	2.40	0.016	83.0	0.001
	HWE >0.05	10	1.67 (1.23, 2.27)	3.26	0.001	81.2	0.001
	HWE <0.05	3	1.12 (0.90, 1.38)	1.02	0.306	0	0.852
GA vs. GG	Overall	13	1.52 (1.33, 1.73)	6.14	0.001	67.9	0.001
	Caucasian	3	1.45 (0.85, 2.49)	1.36	0.173	66.1	0.052
	Asian	11	1.54 (1.34, 1.76)	6.11	0.001	70.6	0.001
	HWE >0.05	11	1.49 (1.24, 1.78)	4.26	0.001	74.6	0.001
	HWE <0.05	3	1.61 (1.42, 1.82)	7.38	0.001	0	0.997
A vs. G	Overall	13	1.29 (1.19, 1.40)	5.97	0.001	65.4	0.001
	Caucasian	3	1.36 (0.96, 1.92)	1.73	0.084	76.5	0.014
	Asian	11	1.28 (1.17, 1.39)	5.66	0.001	64.0	0.003
	HWE >0.05	11	1.30 (1.16, 1.45)	4.57	0.001	73.3	0.001
	HWE <0.05	3	1.25 (1.14, 1.38)	4.81	0.001	0	0.888

OR, odds ratio; CI, confidence interval.

### Publication bias and sensitivity analysis

The Begg’s and Egger’s tests were used to assess the publication bias of the studies involved in this meta-analysis. As shown in Table 3, no obvious publication bias was detected in all models. Moreover, we conducted sensitivity analysis to estimate the influence of individual study on pooled results, and none of the single study affected the pooled OR value, which indicated results were robust.

**TABLE 3:** The effect of publication bias was assessed by Begg’s and Egger’s tests.

Model	Test of Begg (P value)	Test of Egger (P value)
AA vs. GG + GA	0.542	0.297
GA + AA vs. GG	0.393	0.342
AA vs. GG	0.113	0.605
GA vs. GG	0.625	0.584
A vs. G	0.714	0.660

## Discussion

In this meta-analysis, we systematically evaluated the association between PSCA rs2976392 polymorphism and the risk of gastric cancer. By combining data from 13 studies, our results provide robust evidence supporting the significant association of the rs2976392 A allele with increased susceptibility to gastric cancer, particularly in Asian populations. These findings help clarify previously conflicting results and offer new insights into the genetic susceptibility underlying gastric carcinogenesis.

Several previous case-control studies have explored the role of rs2976392 in gastric cancer, but the results have been inconsistent (Kupcinskas et al., 2014; Cai et al., 2017; Dantas et al., 2020). For example, a study by Matsuo et al. (2009) in a Japanese cohort reported a strong correlation between the rs2976392 A allele and an increased risk of stomach cancer, whereas Ou et al. (2010) failed to detect any significant association. Such inconsistencies may arise from limited sample sizes, ethnic heterogeneity, or deviations from Hardy-Weinberg equilibrium. By integrating data from multiple studies and conducting subgroup analyses by ethnicity and HWE status, our meta-analysis overcomes these limitations and reveals a more comprehensive and accurate association pattern. Importantly, we confirmed the presence of a significant association in Asians under all five genetic models, whereas the association in Caucasians was observed only under the homozygous model, suggesting that ethnic-specific genetic background and environmental interactions may modulate the functional relevance of rs2976392.

From a mechanistic perspective, rs2976392 is located in the 5′ untranslated region (UTR) of the PSCA gene, which may influence gene transcriptional activity or mRNA stability (Larios-Serrato et al., 2024). Previous functional studies have indicated that the a allele may lead to reduced PSCA expression, impairing its potential tumor suppressor role in gastric epithelial cells. This is supported by transcriptomic and immunohistochemical studies that demonstrate decreased PSCA levels in gastric cancer tissues compared with adjacent normal mucosa (Chandra et al., 2016; Wu et al., 2020). Reduced PSCA expression may promote abnormal proliferation, loss of cell polarity, and immune evasion, all of which are hallmarks of gastric tumorigenesis (Zhang et al., 2015; Xu et al., 2020). In particular, PSCA has been shown to modulate signaling pathways such as the p38/NF-κB and NF-κB/integrin-α4 pathway, which are involved in cytoskeletal remodeling and motility regulation (Liu et al., 2017; Zhao et al., 2020). Moreover, PSCA downregulation may impair immune surveillance. As a membrane-associated antigen, PSCA may facilitate immune recognition by cytotoxic T cells and natural killer (NK) cells. Loss of PSCA expression on the tumor surface could result in decreased antigen visibility, allowing tumor cells to evade immune detection. Additionally, PSCA downregulation may influence the recruitment or polarization of tumor-infiltrating immune cells, contributing to an immunosuppressive microenvironment that favors tumor progression (Zhang et al., 2007; Ahmad et al., 2009; Wang et al., 2024). These mechanisms collectively underscore the functional significance of PSCA loss in cancer development and highlight its potential as both a diagnostic biomarker and a therapeutic target.

Moreover, our stratified analysis based on HWE status further validated the robustness of our findings. The results showed stable and significant associations across most genetic models, implying that the observed associations are unlikely to be artifacts of population stratification or genotyping errors. Sensitivity analyses also confirmed the stability of the results, as no individual study unduly influenced the pooled effect size, and Begg’s and Egger’s tests did not reveal any publication bias, further reinforcing the reliability of the meta-analysis results.

There are limitations to our meta-analysis that should be noted. First, the majority of the included studies were conducted in Asian populations. In contrast, only a limited number of studies involved Caucasian populations, which may limit the generalizability of our findings across different ethnic groups. Second, some confounding factors, such as *H. pylori* infection status, dietary factors, and histological subtypes of gastric cancer, could not be adjusted for because of a lack of sufficient data. Third, heterogeneity across studies, although reduced through subgroup analysis, may still exist because of uncontrollabl factors, such as geographic distribution, methodological disparities or participant demographics. Fourth, the potential interaction between PSCA rs2976392 and environmental exposure remains insufficiently explored. Elucidating these gene-environment interactions may provide critical insights into the multifactorial nature of GC susceptibility. Future well-designed large-scale studies, including gene environment interaction assessments and adjustments for confounding factors, are needed to further validate our findings. First, large-scale prospective cohort studies involving ethnically diverse populations are needed to validate the association between PSCA rs2976392 and GC risk and to minimize potential biases in case-control designs. Second, functional assays and mechanistic studies should be conducted to elucidate the biological role of PSCA rs2976392 in tumor initiation and progression, including its effects on gene expression, cell signaling, and immune modulation. Finally, given the polygenic characteristics of GC, integrating PSCA rs2976392 and other related SNPs into the polygenic risk model may increase the accuracy of genetic risk prediction and provide a more comprehensive understanding of the genetic factors underlying GC susceptibility.

In conclusion, our meta-analysis provides comprehensive evidence that the PSCA rs2976392 polymorphism is significantly associated with an increased risk of gastric cancer, especially in Asian populations. These results expand our understanding of GC genetics and may provide valuable information for the development of more personalized prevention strategies. Importantly, identification of such risk-associated variants may have important implications for clinical translation. Moreover, PSCA rs2976392 may serve as a biomarker in genetic screening programs, to help identify high-risk individuals.

## Data Availability

The original contributions presented in the study are included in the article/supplementary material, further inquiries can be directed to the corresponding author.
